# T cells and monocytes: A dangerous liaison in adenosine deaminase 2 deficiency

**DOI:** 10.1002/JLB.3CE1021-561R

**Published:** 2022-01-28

**Authors:** Cristina Mesa‐Nuñez, Alessandra Mortellaro

**Affiliations:** ^1^ San Raffaele Telethon Institute for Gene Therapy IRCCS San Raffaele Scientific Institute Milan Italy

Deficiency in adenosine deaminase 2 (DADA2) is a rare autoinflammatory syndrome caused by bi‐allelic loss‐of‐function mutations in the gene encoding ADA2.^1^, ^2^ Disease onset is usually in childhood, and, if undiagnosed or left untreated, patients can die early in life. In this issue of the *Journal of Leukocyte Biology*, Wu and colleagues provide new evidence that T cells from patients with DADA2 show activation of the IFN pathway and may contribute to disease pathogenesis via a cell contact‐dependent mechanism with monocytes.

DADA2 presents with a wide spectrum of clinical manifestations that typically include vasculitis, which can range from mild cutaneous symptoms to life‐threatening stroke and/or brain hemorrhages, immunodeficiency, rheumatological symptoms, and hematologic abnormalities.^1^, ^2^ TNF is a key pathologic factor in the vascular component of DADA2 and anti‐TNF therapy is recommended to reduce the risk of stroke, ameliorate the manifestations of cutaneous and systemic vasculitis, and improve growth and development in patients.^3^ Alongside, allogeneic hematopoietic stem cell transplantation is indicated for patients with cytopenia, bone marrow dysfunction, or immunodeficiency.^4^

The efficacy of anti‐TNF therapy in DADA2 reflects a bigger picture of innate immune dysregulation in the pathology of this disease, which is only beginning to be understood. Studies in patients with rheumatological manifestations of DADA2 reveal an inflammatory syndrome characterized by high levels of pro‐inflammatory cytokines TNF, IL‐6, and IL‐1β.^2^, ^5^ Elevated production of these mediators is thought to be linked with the enhanced pro‐inflammatory activity of M1 Mϕs and impaired development of tissue‐reparative M2 Mϕs seen in cells from patients with DADA2,^2^ which is directly attributed to the absence of ADA2.^5^ Alongside, peripheral blood of patients contains high levels of neutrophil‐expressed transcripts, which may in part be driven by these dysregulated DADA2 Mϕs.^6^ Several studies also point to a pathogenic role for innate immune type I IFNs in DADA2: leukocytes from the peripheral blood of patients with DADA2 exhibit enhanced expression of IFN‐stimulated genes;^7^, ^8^ while single‐cell RNA sequencing (scRNA‐seq) revealed a type I and II IFN signature in monocytes belonging to the classical, intermediate, and non‐classical subtypes.^9^ Based on these observations, it has been proposed that the dysregulated activation of neutrophils and Mϕs might cause activation and damage of endothelial cells, leading to vasculitis and, in more severe cases, to vascular rupture and hemorrhages occurring in patients with DADA2.

DADA2 also presents with immunodeficiency. Early reports showed normal frequencies and numbers of T cells in patients^2^ but reduced class‐switched memory B cells and plasmablasts, and panhypogammaglobulinemia,^10^ suggesting a defective B‐cell terminal differentiation. Indeed, an arrest of B‐cell development was observed at the transition from pro‐B to pre‐B cells in the bone marrow of ten patients with DADA2.^11^ However, more recent analyses with larger groups of patients revealed an increased frequency and an absolute number of naïve CD4^+^ T cells, while the central and effector memory T‐cell compartment (CD4^+^ and CD8^+^) was significantly smaller than in healthy controls (HDs).^10^, ^11^ Patients’ T cells also showed a more exhausted and senescent phenotype.^11^ Interestingly, an expansion of functionally‐impaired circulating follicular helper T cells was reported in a pediatric cohort, which may also explain the reduced B‐cell functionality seen in DADA2.^10^ These reports showing profound differences in the T‐cell compartment in DADA2 has led to multiple questions on the potential role of T cells in the disease: what are the detailed phenotypes of the altered T‐cell populations, how are T‐cell functions affected by DADA2, and do these T cells recognize any clinically relevant Ags in the disease?

In this issue of the *Journal of Leukocyte Biology*, Wu et al. conducted deep transcriptional profiling of CD4^+^ and CD8^+^ T cells isolated from the peripheral blood of 10 patients with DADA2 under TNF therapy, and 5 age‐ and sex‐matched HDs using scRNA‐seq coupled with single‐cell TCR sequencing (scTCR‐seq). This approach revealed similar clustering of naïve, central memory, and effector memory CD4^+^ and CD8^+^ T‐cell subsets in treated patients and HDs. Moreover, TCR sequencing of single cells detected low‐level expression of the majority of TCR and widespread multi‐clonality; thus, no evidence of clonal expansion in patients with DADA2. However, despite these similarities, when the authors delved deeper and investigated the gene expression profile of patients’ T cells, they uncovered significantly higher expression levels of genes involved in IFN and inflammatory response pathways in both CD4^+^ and CD8^+^ T‐cell subsets, as well as genes related to the PI3K‐AKT‐mTOR signaling pathway, apoptosis, UV response, MYC targets, innate immune response, Ag processing, and Ag presentation. Together, these data paint a picture of generalized dysregulation of signaling in DADA2 T cells, despite the broadly normal T‐cell repertoire.

Given the profound disturbance of myeloid cell functions seen in patients with DADA2, the authors went on to ask whether the transcriptomic difference in T cells could be due to altered crosstalk with monocytes. Wu et al. exploited scRNA‐seq data generated from the same patients’ T cells and monocytes in their previous study^9^ to infer ligand‐receptor and cell‐cell interactions between T cells and monocytes. They first identified underlying cellular networks that may be involved in DADA2 pathogenesis, finding that there are more ligand‐receptor interactions between monocytes and CD8^+^ T cells than between monocytes and CD4^+^ or CD4^+^CD8^+^ T cells. They also found that several ligand‐receptor interactions are relatively over‐represented in patients with DADA2, and some, such as that between CCL4 and CCR8, is uniquely present between CD8^+^ T cells and monocytes in this condition. The authors also used their monocyte/T cell dataset to interrogate the downstream transcriptional changes induced by key ligand‐receptor interactions: of note, they identified signal transducer and activator of transcription 1 (STAT1) as an important hub for IFN signaling in both T cells and monocytes. Taken together, the molecular connections between T cells and monocytes in patients with DADA2 warrant further investigation and could feasibly represent a route to pinpointing new potential therapeutic targets for the treatment of this disease.

Overall, Wu et al. provide the first evidence that, although patients’ T cells do not show evident abnormalities in terms of subsets, clonality, or TCR usage, they may still be involved in DADA2 pathogenesis by contributing to aberrant monocyte activation dominated by an IFN response. However, some limitations of the study deserve mention. First, the monocyte‐T cell interactions are mostly speculative as they are derived from in silico predictions. Therefore, validation studies are extremely important to establish a link between aberrant T cell‐monocyte crosstalk and disease pathogenesis. Second, all patients were under anti‐TNF therapy at the time of the evaluation. TNF neutralization might have influenced the profiling of T cells and monocytes. Therefore, the data ought to be confirmed in a pretreatment cohort. Lastly, earlier observations that the elevation of the IFN signature seems to correlate with disease severity^8^ suggest that IFN dysregulation may be central in DADA2 pathogenesis. However, some pieces of evidence point to a more general dysregulated inflammatory response as a cause of the clinical features of DADA2: in a small cohort of patients, treatment with the TNF inhibitor etanercept significantly reduced the IFN signature,^8^ suggesting that IFN pathway up‐regulation in DADA2 may generally depend on TNF. Moreover, the fact that TNF signaling enhances IFN induction has been reported in other immune‐mediated inflammatory diseases, such as rheumatoid arthritis.^12^ However, evidence that IFN signaling can also induce TNF production similarly exist: indeed, the IFN‐STAT1 axis is a potent inducer of M1 Mϕ polarization. Therefore, more investigations are needed to understand whether the reciprocal cross‐regulation of TNF and IFN might have clinical relevance in DADA2.

In conclusion, the current study by Wu and colleagues reveals an important crosstalk between T cells and monocytes that contributes to driving up‐regulation of STAT1‐mediated IFN responses in DADA2. Leading on from this work, urgent investigations should aim to pinpoint the exact molecular mechanism underlying the activation of the IFN pathway in DADA2 and how this pathway ties in with neutrophil and Mϕ defects. A better characterization of the triggers and pathways involved in the induction of type I IFN in specific cell types of immune and non‐immune origin will be essential to understand its action.

## AUTHORSHIP

C.M.N. prepared the graphical abstract and edited the manuscript; A.M. wrote the manuscript.
